# High Prevalence of Potential Molecular Therapeutic Targets in Poorly Differentiated Thyroid Carcinoma

**DOI:** 10.1007/s12022-025-09883-y

**Published:** 2025-10-22

**Authors:** Vanessa Zambelli, Giulia Orlando, Marta Fornaro, Giulia Vocino Trucco, Ida Rapa, Francesca Napoli, Susanna Cappia, Lorenzo Daniele, Simonetta Piana, Mauro Papotti, Marco Volante

**Affiliations:** 1 Department of Oncology, University of Turin, at San Luigi Hospital, Regione Gonzole 10, Orbassano, Turin 10043 Italy; 2 Department of Oncology, University of Turin, at Città Della Salute E Della Scienza Hospital, Turin, Italy; 3 Pathology Unit, San Luigi Hospital, Orbassano, Turin Italy; 4 Pathology Unit, Mauriziano Hospital, Turin, Italy; 5 Pathology Unit, Azienda Usl-IRCCS Reggio Emilia, Reggio Emilia, Italy

**Keywords:** Poorly differentiated, Thyroid, Carcinoma, Molecular, Biomarkers

## Abstract

Poorly differentiated thyroid carcinoma (PDTC) is a rare thyroid cancer with aggressive clinical course and peculiar clinical/pathological characteristics but lacking effective therapeutic options, when surgery is not curative. We aimed at the molecular characterization of PDTC with a specific focus on the identification of potential therapeutic targets. A series of PDTC cases was selected from a multi-institutional network. Fifty-nine samples underwent wide targeted DNA and RNA next-generation sequencing (NGS) testing and immunohistochemical analysis for mismatch repair (MMR) proteins. Gene fusion analysis was enriched by 25 additional samples. Prevalence of MMR protein loss was 11.9%. The most prevalent mutations were in *NRAS* (25%) and *TP53* (25%), mutually exclusive. *TERT* promoter (*TERTp*) mutations were detected in 19.6% of cases (10/51). *NRAS*-mutated cases were enriched for mutations in genes belonging to the same pathway. *TP53*-mutated samples lacked *TERTp* co-mutations, but were associated with mutations in *PTEN* and in genes related to MMR system and/or loss of MMR proteins. *TERTp* mutations were the most prevalent alterations (28%, 7/25) in a third group that lacked *NRAS* or *TP53* mutations. Four cases harbored gene fusions, including two cases harboring the *TBL1XR1::PIK3CA* fusion that has never been reported in thyroid cancer, so far. In conclusion, PDTC may be genomically segregated in subgroups with specific molecular characteristics. Overall, targetable gene fusions have a prevalence of 9% (4/42). Moreover, 47% of cases are potential candidates for individualized target therapies since they harbor mutations in genes coding for potentially targetable molecules and/or have defects in the MMR system.

**Supplementary Information:**

The online version contains supplementary material available at https://doi.org/10.1007/s12022-025-09883-y.

## Introduction

Poorly differentiated thyroid carcinoma (PDTC) represents a heterogeneous group of aggressive thyroid neoplasms that has been a subject of discussion since its original description in the early 1980s. Subsequent studies have shown that PDTC accounts for 2–15% of all thyroid carcinomas [1] and exhibits an intermediate prognosis between differentiated thyroid carcinomas of follicular cell origin—including papillary and follicular carcinomas—and anaplastic thyroid carcinoma, with a 5-year disease-specific survival rate of 66% [2].

Poorly differentiated thyroid carcinoma was first introduced as a distinct thyroid cancer subtype in the 2004 WHO Classification of Tumors. In 2007, a diagnostic algorithm was proposed in the so-called Turin consensus, which incorporated specific architectural patterns (solid, insular, and/or trabecular growth) with high-grade features, such as increased mitotic activity and/or presence of tumor necrosis [3].

An alternative diagnostic framework, proposed by the Memorial Sloan Kettering Cancer Center (MSKCC), defined PDTC as a group of aggressive follicular cell–derived thyroid carcinomas characterized solely by high-grade features, irrespective of architectural pattern [4]. Subsequent studies confirmed that both approaches are able to recognize follicular cell–derived thyroid cancers bearing an intermediate prognosis [5, 6]. These findings paved the way for the current 2022 WHO classification, which identifies a broader category of high-grade follicular cell–derived non-anaplastic thyroid carcinomas encompassing two distinct histological entities: PDTC, as defined by the Turin consensus criteria, and high-grade differentiated thyroid carcinoma [7].

Poorly differentiated thyroid carcinoma may arise de novo or progress from differentiated carcinomas of thyroid follicular cell origin. However, the de novo pathogenetic hypothesis is mainly based on the description of PDTC cases lacking an associated differentiated thyroid carcinoma component, but this finding may simply reflect sampling limitations and/or tumor overgrowth. By contrast, molecular data are more consistent with a general model of multi-step progression from differentiated to poorly differentiated and eventually to anaplastic carcinoma, with somatic genetic alterations that can be categorized as “early” and “late” events [8].

“Early” driver changes are mostly *RAS* and *BRAF* p.V600E mutations. Moreover, similarly to what is described for anaplastic thyroid cancer, the most frequent “late” changes are *TP53* and *TERT* promoter (*TERTp*) mutations or alterations of the PI3K/PTEN/AKT pathway. Gene fusions are expected to be rare, but PDTC histology is enriched in cases harboring such alterations [9].

The heterogeneity of classification criteria has been a major bias in terms of the definition of the main molecular characteristics of PDTC. A seminal study depicted in detail the molecular landscape of PDTC, but at the same time highlighted how the two different classification approaches were interfering with the molecular mapping [10]. In fact, between the two main molecular subgroups identified, the *BRAF*-like group was dominated by MSKCC-classified cases and the *RAS*-like group was dominated by Turin Consensus–classified cases [10]. Indeed, subsequent reports were supportive of the molecular diversity of genomic alterations between PDTC and high-grade differentiated thyroid carcinoma as proposed in the new WHO classification [11]. Therefore, most of the available literature on the genomic landscape of PDTC is influenced by non-homogeneous inclusion criteria, by the relatively small sample size of analyzed series, and by a pathogenetic rather than clinically driven approach. All these factors influence the relative prevalence of molecular alterations detected and their integration with pathological and clinical data.

In terms of therapeutic strategies, unlike papillary and follicular thyroid carcinomas, PDTC therapy is not standardized due to the rarity of the disease and the heterogeneity of inclusion criteria in the few clinical studies available. Radioiodine responsiveness of PDTC after surgery is variable, possibly as the result of intra-tumor heterogeneity and coexistence of well and less well-differentiated tumor components [12]. Treatments using novel therapeutics have been proposed in thyroid cancer with no response or progression after radioiodine treatment [13]. However, no robust data are available in PDTC, with special reference to the prevalence of alterations in potential targets or to the real clinical benefit of therapeutic targeted approaches.

Based on the above, there is a strong need to identify novel strategies that might lead to a better personalized approach and individualization of the therapeutic strategies in PDTC. Therefore, the aim of this study was to characterize a series of PDTC, homogeneously coded following the Turin criteria as proposed for this group in the current WHO classification, by means of a multimodal molecular approach with the objective of identifying the prevalence and potential clinical usefulness of molecular targets for therapy. We decided to restrict the analysis to the PDTC subtype because homogeneous criteria for its definition were claimed to provide a robust and reliable group of tumors and—last but not least—because these particular tumors are more common in alpine/mountain areas including our country, and their molecular characterization is less established in the literature as compared to high-grade differentiated thyroid carcinoma.

## Materials and Methods

### Patient and Tissue Samples

Fifty-nine samples of PDTC were selected from the files of the Pathology Units at “San Luigi” and “Città della Salute e della Scienza” Hospitals and tested for the presence of mismatch repair defects and for DNA and RNA alterations through a wide targeted NGS approach. Due to the high number of failures in RNA analysis (see below), 25 additional PDTC samples from Mauriziano (Turin) and Reggio Emilia Hospitals were added to RNA analysis. All samples were formalin-fixed and paraffin-embedded (FFPE) surgical materials, retrieved from years 1993 to 2022. For all enrolled cases, histological slides were re-assessed by a pathologist (MV) to confirm the diagnosis following diagnostic criteria for PDTC proposed by the Turin Consensus [3] and embraced by the current WHO classification [7]. Major clinical and pathological data were collected and included sex, age, presence of predominant oncocytic features (> 75% of the tumor), pTN stage according to AJCC system 8th edition, tumor size, presence of necrosis, presence of angioinvasion, mitotic index in 2 mm^2^, presence of recurrences/metastases, site of metastases, and patient outcome in terms of absence or presence of disease in patients alive, or the cause of death. The study was approved by the local Ethical Committee (#610, on December 20, 2017) and conducted in accordance with the principles set out in the Declaration of Helsinki. Considering the retrospective nature of this research protocol and that it had no impact on patients’ care, no specific written informed consent was required.

### Nucleic Acid Extraction and Sample Quality Control

Genomic DNA and RNA were extracted from FFPE tumor material. Enrichment of tumor cells was achieved by manual microdissection under light microscopy from one to ten sections for each case as previously described [14].

The selected material was extracted using Maxwell RSC DNA FFPE kit (Promega Corporation, Madison, WI, USA, CN: AS1450) and Maxwell RSC RNA FFPE kit (Promega Corporation, Madison, WI, USA, CN: 14402) according to the manufacturer’s instructions. Nucleic acids were quantified on Quantus fluorometer (Promega Corporation, Madison, WI, USA) using Quantifluor DNA System (Promega Corporation, Madison, WI, USA, CN: E4871) and Quantifluor RNA System (Promega Corporation, Madison, WI, USA, CN: E3310) following the manufacturer’s instructions.

DNA quality was evaluated with real-time PCR of *EGFR* Exon2 amplification through Rotor-Gene Q (Qiagen, Hilden, Germany) Real Time PCR instrument, and the following primers were used for *EGFR*: EGFRex2b Fw (5′-GAAGATCATTTTCTCAGCCTCCA-3′) and EGFRex2b Rw (5′-AGGAAAATCAAAGTCACCAACCT-3′) (Diatech Pharmacogenetics, Jesi, Ancona, Italy). RNA quality was evaluated with real-time PCR with beta-actin amplification through Rotor-Gene Q (Qiagen, Hilden, Germany) Real Time PCR instrument, and the following primers were used for B-ACT: BACT Fw (5′-CCTTCCTGGGCATGGAGTCTTG-3′) and BACT Rw (5′-GGAGCAATGATCTTGATCTTC-3′).

### Analysis of Mismatch Repair Status

The expression of mismatch repair (MMR) proteins was tested using immunohistochemistry in an automated system (Dako Omnis, Dako, Agilent) using the following antibodies (all from Dako): MLH1 (clone ES05, CN:GA079), MSH2 (clone FE11, CN:GA085), MSH6 (clone EP49, CN:GA086), and PMS2 (clone EP51, CN:GA087). Loss of nuclear expression for paired proteins (MLH1 and/or PMS2 or MSH2 and/or MSH6) was considered as altered expression pattern. Cases with an altered pattern were also tested for the presence of microsatellite instability (MSI) using genomic DNA extracted as described above. Since thyroid cancer–specific panels are not commercially available, all cases were analyzed using a kit clinically approved for colon and endometrial cancer (EasyPGX ready MSI KIT CE IVD, Diatech Pharmacogenetics, CN:RT033) that includes the following markers: BAT25, BAT26, NR21, NR22, NR24, NR27, CAT25, and MONO27. Bioinformatic analysis was carried out through the software for data exportation Agilent Aria Software v1.4, and data analysis was performed with EasyPGX Analysis Software v3.0. Results are expressed as microsatellite stable (MSS), low microsatellite instability (MSI-low), and high microsatellite instability (MSI-high).

### Next-generation Sequencing

Library preparation was carried out automatically using the DNA and RNA Oncomine Comprehensive Assay v3 (Thermo Fisher Scientific, Waltham, MA, USA, CN: A36111) using a total from 10 to 40 ng input DNA and RNA in an Ion Chef System (Thermo Fisher Scientific, Waltham, MA, USA) following the manufacturer’s instructions. The Oncomine Comprehensive Assay v3 (Thermo Fisher Scientific, Waltham, MA, USA) comprises a DNA panel which was designed to interrogate hotspot mutations (#87), full exon coverage (#48), and copy number variations (#43) and an RNA panel which was designed to interrogate fusion drivers (#51) (SupplementaryTable 1). This panel can identify current actionable genetic variants and potential future targets for personalized therapy.

The prepared libraries were clonally amplified onto Ion Sphere Particles (ISP) using emulsion PCR in an Ion Chef System (Thermo Fisher Scientific, Waltham, MA, USA) according to the manufacturer’s instructions. Enriched ISPs were loaded onto 540 chips accommodating eight DNA samples and eight RNA samples on a single chip and sequencing on the Ion Torrent S5 Prime Studio (Thermo Fisher Scientific, Waltham, MA, USA), according to the manufacturer’s instructions.

### DNA Data Analysis

Analysis was carried out using Ion Torrent Suite Browser version 5.16 (Thermo Fisher Scientific, Waltham, MA, USA) and Ion Reporter version 5.16 (Thermo Fisher Scientific, Waltham, MA, USA). The Torrent Suite Browser was used to perform initial quality control including chip loading density, median read length, and number of mapped reads. The Coverage Analysis plugin was applied to all data and used to assess amplicon coverage for regions of interest.

The Ion Reporter suite (Thermo Fisher Scientific, Waltham, MA, USA) was used to filter out known polymorphic variants. The variants were annotated by genetic databases: the Single Nucleotide Polymorphism Database (dbSNP) (http://www.ncbi.nlm.nih.gov/projects/SNP/), Catalogue of Somatic Mutations in Cancer (COSMIC) (http://cancer.sanger.ac.uk/cancergenome/projects/cosmic/), and ClinVar database (http://www.ncbi.nlm.nih.gov/clinvar/).

Variants with altered allele depth ≤ 100 base coverage and a variant allelic frequency ≤ 5% were eliminated from the analysis. Identified variants were checked for correct nomenclature using Alamut Visual Plus (Interactive Biosoftware; Sophia Genetics). Any discrepancies in variant identification, between Ion Reporter and Alamut, were validated manually using the Integrative Genomics Viewer [15].

Variants were annotated following ACGM guidelines [16] and the search engine VarSomePremium.com [17].

The prediction of functional effects of the variants that were found as Variants of Uncertain Significance (VUS) was assessed with 13 in silico tools (Align GVGD [http://agvgd.hci.utah.edu/agvgd_input.php], Mutation Taster [https://www.mutationtaster.org], Provean [http://provean.jcvi.org], SIFT [https://sift.bii.a-star.edu.sg], Grantham [https://ionreporter.thermofisher.com], Polyphen2 [https://ionreporter.thermofisher.com], DANN [https://varsome.com], FATHMM-MKL [https://fathmm.biocompute.org.uk], LRT [https://sites.google.com/site/jpopgen/dbNSFP], Meta-RNN [http://www.liulab.science/metarnn.html], MutPred [http://mutpred.mutdb.org], Mutation Assessor [http://mutationassessor.org/r3], and REVEL [https://labworm.com/tool/revel]) (Supplementary Table 2). Each tool had its own threshold, giving for each score a prediction of tolerated or damaging. VUS was qualified as damaging when the sum of all in silico tools that resulted damaging was higher than 7 [18]. The missense variants called as both benign and tolerated were excluded, as well as variant shaving a frequency higher than 1% in all populations from the 1000 Genomes data. Synonymous mutations were excluded from the analysis. RNA Data analysis was carried out using Ion Torrent Suite Browser version 5.16 (Thermo Fisher Scientific, Waltham, MA, USA) and Ion Reporter version 5.16 (Thermo Fisher Scientific, Waltham, MA, USA).

### Sanger Sequencing

To validate *TERTp* mutations that are difficult to detect in NGS analysis as they are intronic, we performed Sanger sequencing analysis on all 59 cases tested for DNA genomic alterations in NGS. *TERTp* region was sequenced for the detection of the two mutations C228T and C250T. Target region was amplified by conventional PCR with the following primes: *TERT* Fw (5′AGTGGATTCGCGGGCACAGA-3′) and *TERT* Rw (5′-CAGCGCTGCCTGAAACTC-3′). A first step with Uracil-DNA Glycosylase (Thermo Fisher Scientific, Waltham, MA, USA) was performed on all samples, following manufacturer’s instructions. Then, the PCR run in 50-µL reactions with 25 µL of 2X Platinum SuperFi II PCR Master Mix (Thermo Fisher Scientific, Waltham, MA, USA, CN:12361010), 5 µM of each primer, and 10 µL of gDNA. The amount of gDNA for each PCR varies from 5 to 100 ng, depending on the sample’s quality. PCR conditions consist of one cycle of 98 °C for 1 min; 3 cycles of 98 °C for 30 s, 62 °C for 30 s, and 72 °C for 45 s; followed by 35 cycles of 98 °C for 30 s, 60 °C for 30 s, and 72 °C for 45 s; and final extension at 72 °C for 5 min. Resulting amplicons were visualized in 2% agarose gels and verified to have the expected size of 193 bp. *TERTp* sequences were generated by Sanger sequencing, and sequencing was performed at Eurofins Genomics (Ebersberg, Germany); all samples were sequenced in both directions.

#### Fluorescence In Situ Hybridization

To validate the *TBL1XRA::PIK3CA* fusion, a FISH approach was applied on 4-µm-thick sections from FFPE material using a *TBL1XR1/PIK3CA* probe set (Empire Genomics, New York, USA, CN: TBL1XR1-PIK3CA-20-GROR) following manufacturer instructions. The two cases positive in RNA NGS analysis and two cases negative, randomly selected from the series, were tested. *TBL1XR1/PIK3CA* probe set consisted of DNA labeled in Spectrum Green and Spectrum Orange. The DNA probe set hybridizes to chromosome 3q26.32 (Green) and 3q26.32–q26.33 (Orange) in interphase nuclei. The presence of two green and red separated signals were considered as normal pattern, while altered partner was characterized by fused signals (yellow) and/or with multiple red and green signals without fusion signal. The sections were examined with an Olympus BX61 fluorescence microscope (Olympus Corporation, Tokyo, Japan) equipped with a triple-pass filter (DAPI/Green/Orange; Vysis, Downers Grove, IL,USA) with CytoVision software version 7.6 (Leica Biosystems, Buffalo Grove, IL, USA).

#### Statistical Analyses

Pathological features and immunohistochemical and molecular results were correlated to clinical variables, using appropriate statistical tests (Fisher’s or chi-square for qualitative and *t* Student’s test for quantitative parameter correlation) and univariate analyses (log-rank test) of both disease-free interval (from the date of diagnosis to first metastasis/recurrence) and disease-specific survival (from the date of diagnosis to death if related to the disease). All statistical analyses were performed using Graph Pad Prism 9.4.1 software.

## Results

### Clinicopathological Characteristics

Eighty-four cases were selected for the study, including 59 cases collected at the “San Luigi” and “Città della Salute e della Scienza” Hospitals for DNA and RNA testing, and 25 additional cases selected for RNA analysis enrichment at the Reggio Emilia and Mauriziano Hospitals.

The series included 47 females and 37 males. Mean age was 62.8 years (range 27–87 years). Information related to tumor size and pathological stage were available for 68 patients. Mean size was 6.0 cm (range 1.5–17). Pathological T stage distribution was pT1b in 1.5% (1/68), pT2 in 13.2% (9/68), pT3a in 70.6% (48/68), and pT4a in 14.7% (10/68). Positive N stage was observed in 29.4% of cases (20/68).

Mitotic index, presence of necrosis, and presence of angioinvasion were reassessed (by MV) on available histological material from all 84 samples. Mean mitotic index in 2 mm^2^ was 4.1 (range 1–20). Necrosis was present in 90.5% of cases (76/84). Angioinvasion was present in 100% of cases (84/84). Patients’ outcome data were available in 60 cases. Twenty cases (33.3%) were alive with no evidence of disease or died for causes unrelated to disease, whereas 66.7% (40/60) were alive with disease or died of the disease at the last follow-up.

### Mismatch Repair Status

**Fig. 1:**
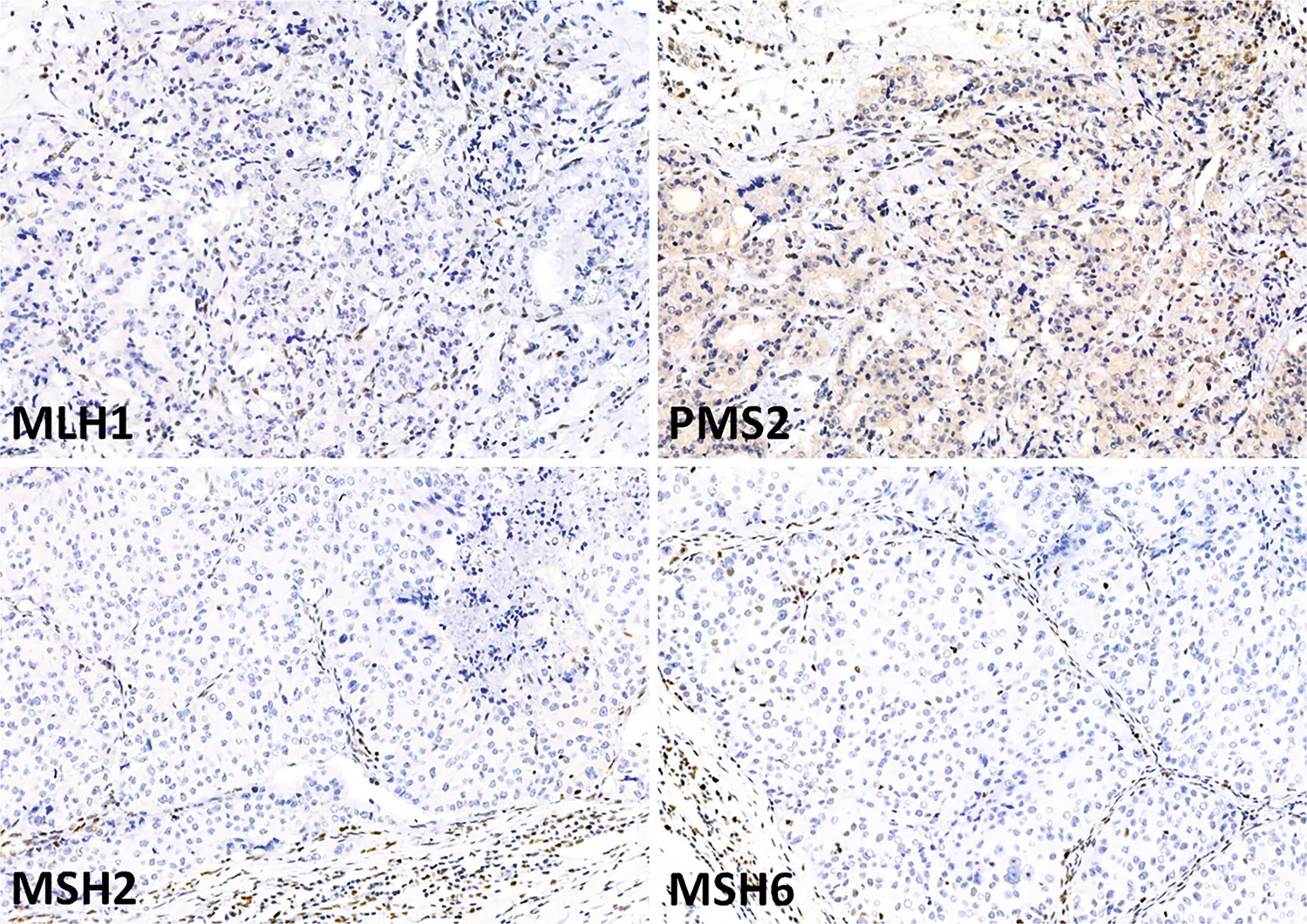
Representative images of MLH1, PMS2, MSH2, and MSH6 altered expression, with negative nuclear staining in tumor cells and positive nuclear staining in non-neoplastic elements (mostly endothelial cells and lymphocytes)

All samples were adequate for analysis, with a reliable reactivity of the tested markers in positive control cells within the tissue sections. Seven out of 59 cases (11.9%) had an altered MMR protein pattern (Fig. 1). In particular, four cases had MSH2-MSH6 loss, one sample MLH1-PMS2 loss, one sample MSH6 loss, and one sample PMS2 loss. However, molecular analysis aimed at testing the presence of instability in the microsatellite loci covered by the EasyPGX panel failed to detect an altered pattern in cases with loss of MMR protein expression.

### Molecular Profiling

Fifty-one over 59 PDTC samples (86.4%) were suitable for DNA NGS analysis. The eight cases with inadequate DNA for NGS analysis had an age of blocks ranging from 2002 to 2016. Mean age in years of blocks in adequate and inadequate samples was 14 and 11, respectively (*p* = 0.36).

**Fig. 2:**
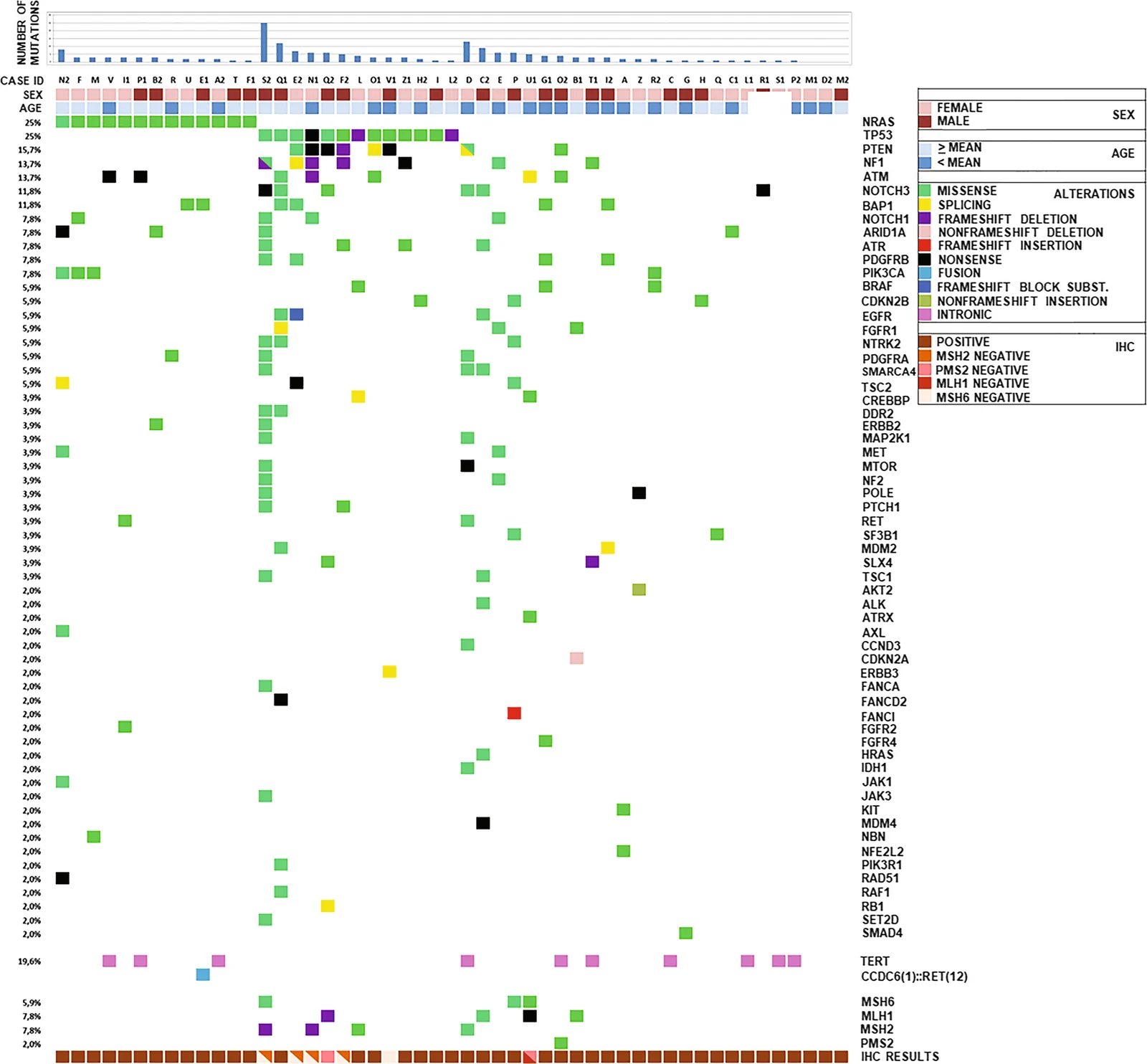
Heat map of genomic DNA alterations detected in 51 PDTCs

Genomic alterations found in the series are summarized in Fig. 2. Details in genomic DNA alterations, as well as RNA fusions and CNV detected in the series, are reported in Supplementary Table 3.

The number of overall mutations per case ranged from 1 to 25 (mean 3.7). The three main molecular groups were represented by *NRAS*-mutated cases (25.5%, 13/51), *TP53*-mutated cases (25.5%, 13/51), and non-*RAS*/non-*TP53* mutated cases (49%, 25/51). *NRAS* and *TP53* mutations were mutually exclusive. *TERTp* mutations were detected in 19.6% of cases (10/51; 9/10 C228T [c.−124 C > T] and 1/10 C250T [c.−146 C > T]). All *TERTp* mutations detected through NGS analysis were confirmed by means of Sanger sequencing. No additional mutations in *TERTp* were detected by Sanger sequencing analysis in NGS negative cases, with an overall concordance between the two methods of 100%.

Mutations in MMR genes were detected in 19.6% of cases (10/51). Mutational profile in MMR genes was concordant in three samples with protein loss at immunohistochemistry, including two cases with *MSH2* mutation (one with and one without associated *MSH6* mutations) and one case with *MLH1* mutation. One additional case harbored *MLH1* mutation but loss of *PMS2* protein, only. In the remaining three cases with altered expression of MMR proteins, no mutations in MMR genes were detected. Six additional cases harbored mutations in MMR genes (two *MLH1*, two *MSH2*, one *MSH6*, and one *PMS2*) with no loss of MMR protein expression.

Other frequent alterations, in order, were in *PTEN* (15.7%), *NF1* (13.7%), *ATM* (13.7%), *NOTCH3* (11.8%), and *BAP1* (11.8%). Three cases were wild type for all genes included in the NGS panel.

*NRAS*-mutated and *TP53*-mutated cases showed different molecular characteristics. Mean number of alterations was higher in *TP53*-mutated cases (5.8 mutations/case) rather than in *NRAS*-mutated cases (2.8 mutations/case). *PIK3CA* and *TERTp* were the most prevalent co-mutated genes (three cases, each, mutually exclusive) in *NRAS*-mutated cases. *TP53*-mutated samples lacked *TERTp* co-mutations but were significantly associated with mutations in *PTEN* (46.1%, 6/13; *p* = 0.024 as compared with the other molecular subgroups) and in genes related to MMR system and/or loss of MMR proteins (53.8%, 7/13 cases; *p* = 0.005 as compared with the other molecular subgroups). Overall, most co-occurring mutations in *TP53*-mutated cases, as compared to *NRAS*-mutated cases, were mutually exclusive (SupplementaryFig. 1). However, gene alterations belonging to the *RAS*-like molecular class (i.e., *BRAF* non-V600E and *PTEN* mutations) occurred as co-mutations in the *TP53*-mutated group. A third heterogeneous group (25 cases) lacked *NRAS* or *TP53* mutations and had a low mean number of alterations (2.7 mutations/case). *TERTp* mutations were the most prevalent alterations in this group (28%, 7/25, not reaching statistical significance as compared to the two other molecular subgroups). One case with *HRAS* mutation was aggregated within this group because of the co-presence of different other mutations and a low allelic frequency (14%). Copy number variations were not detected.

Twenty-nine out of 59 cases were adequate for RNA NGS analysis (49.1%). Due to this high rate of failure, 25 additional cases were included. Overall, 84 samples were tested, with 42 cases passing quality controls for analysis (50%). Mean age of blocks in adequate and inadequate samples was 11 and 12, respectively (*p* = 0.38).

**Fig. 3:**
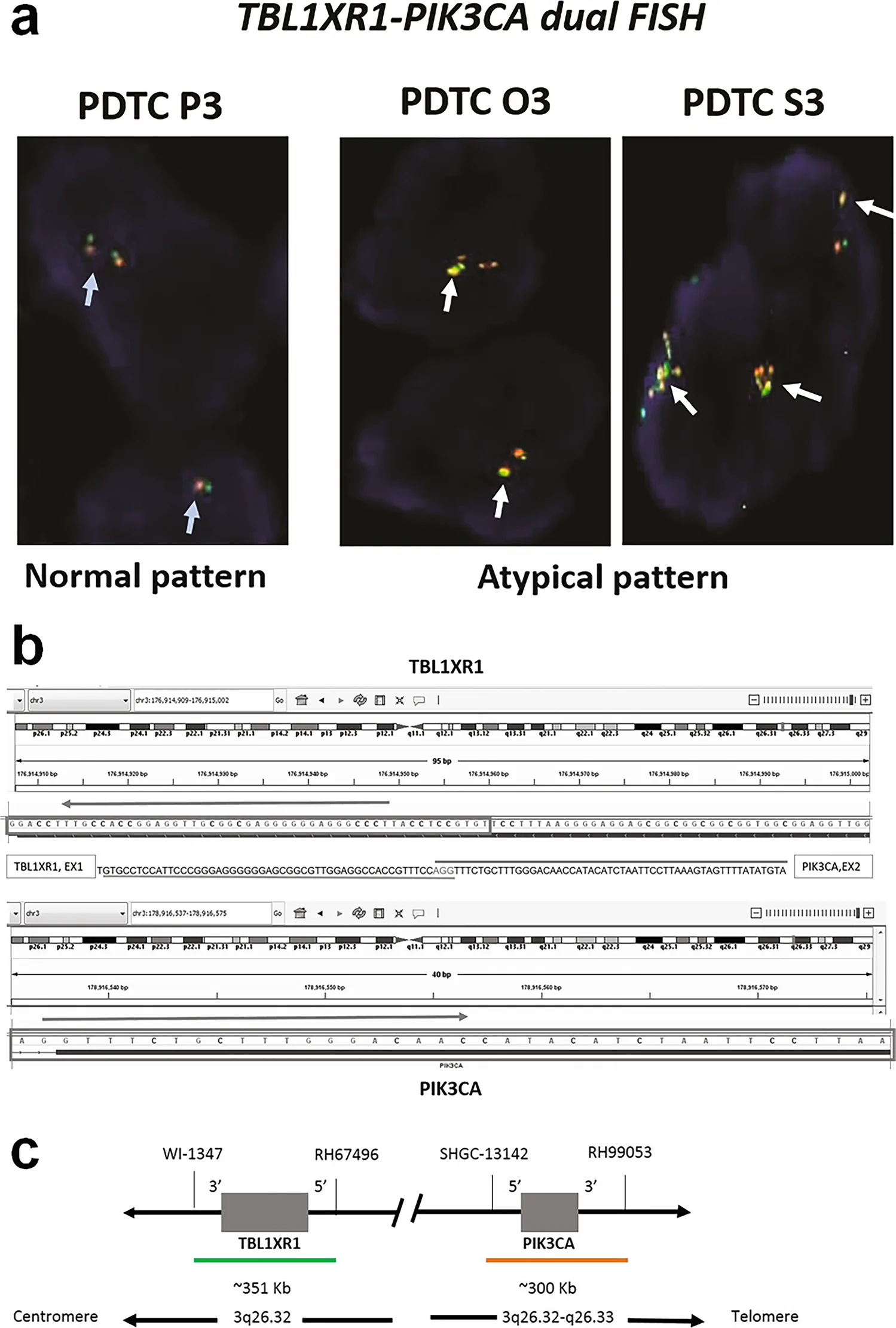
**a** Dual FISH analysis showing abnormal pattern in two cases with *TBL1XR1::PIK3CA* fusion and normal pattern in a wild type case; **b** IGV image of genes involved in fusion *TBL1XR1::PIK3CA* (the first exon of *TBL1XR1* is fused to the second exon of *PIK3CA* by inversion) and overlap point between *TBL1XR1* and *PIK3CA* sequences (3 Gy nucleotides, AGG); **c** schematic illustration of the dual DNA probe set employed to detect the presence of the *TBL1XR1::PIK3CA* fusion

Chromosomal rearrangements involving genes known to be translocated in thyroid cancer were found in two samples, including one case with *RET* rearrangement involving the common *RET* partner *CCDC6* and one case with the *PAX8::PPARG* fusion. Two other cases harbored a *TBL1XR1::PIK3CA* fusion (Fig. 3). In the remaining 38 samples no gene fusions were detected. The presence of the *TBL1XR1::PIK3CA* fusion was associated with an altered pattern by FISH in both the two positive cases, whereas fusion-negative samples showed the expected non-altered pattern.

### Clinical and Pathological Correlations

**Fig. 4:**
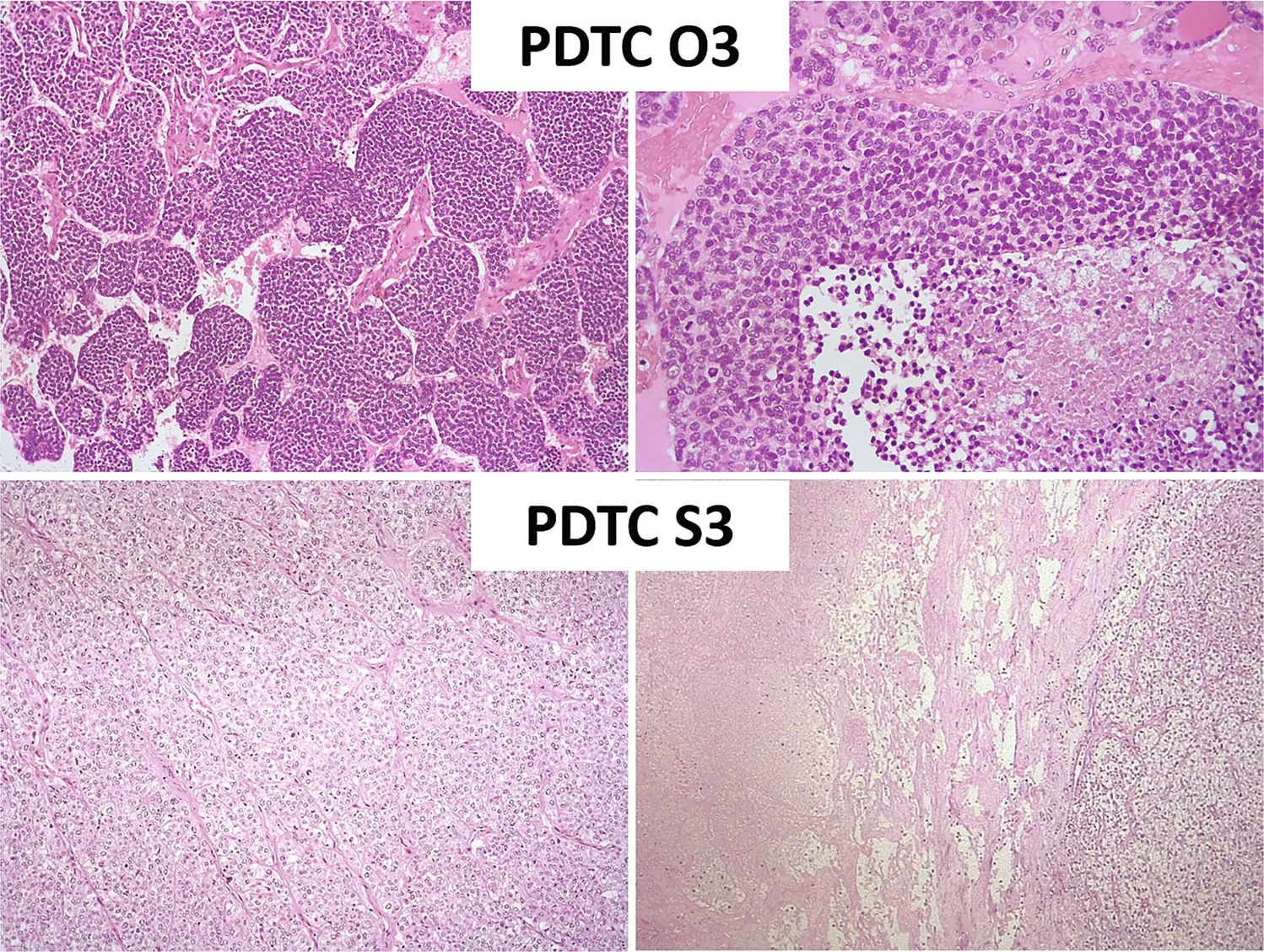
Pathological features of the two cases harboring the *TBL1XR1-PIK3CA* fusion (all hematoxylin and eosin stainings). PDTC case #O3 displayed an insular growth pattern (left panel) and foci of comedo-necrosis (right panel). PDTC case #S3 had a solid growth (left panel) and extensive areas of necrosis (right panel)

The most prevalent molecular findings in our series were compared with major clinical and pathological characteristics (Table 1). Cases showing MMR protein loss and *TERTp*-mutated cases were not associated with significant clinical or pathological characteristics in our series. The three distinct molecular subgroups did not show any significant association with clinical or pathological parameters, except for a higher prevalence of PDTC with predominant oncocytic features in the *TP53*-mutated group. Moreover, although not reaching statistical significance, *TP53* and *TERTp* mutated cases had a higher prevalence of adverse events as compared with *NRAS*-mutated cases. The two cases with the *TBL1XR1::PIK3CA* fusion had conventional pathological features with no peculiar findings (Fig. 4). Survival data were available in 47 cases. Median survival times were calculated in the three major subgroups. Median disease-free survival was 17, 15, and 64 months in *NRAS*-mutated, *TP53*-mutated, and non-*NRAS*/non-*TP53* mutated cases, respectively, with a trend to statistical significance with log-rank test (*p* = 0.079). Median disease-specific survival was 145, 111 and 274 months in *RAS*-mutated, *TP53*-mutated, and non-*NRAS*/non-*TP53* mutated cases, respectively, without a statistically significant difference.

**Table 1:** Clinicopathological correlations according to molecular subgroups

Parameter	MMRp	MMRd	*p* Value	*NRAS* mutated group	*TP53* mutated group	*non-RAS/non-TP53* mutated group	*p* Value	*TERTp* wt	*TERTp* mutated	*p* Value
Sex (M/F)	18/26	4/3	0.45	5/8	6/7	11/14	0.92	18/23	4/6	0.99
Age (median, range)	62	67	0.56	68	67	61	0.33	65	61	0.40
Predominant oncocytic features (yes/no)	21/23	6/1	0.10	3/10	11/2	13/12	0.007	20/21	7/3	0.30
pTstage (pT1–2/pT3–4) (3 cases missing)	8/33	1/6	0.99	2/11	2/10	5/18	0.87	8/30	1/9	0.42
pN stage (pN0-NX/pN +) (3 cases missing)	25/16	3/4	0.43	8/5	6/6	14/9	0.79	21/17	7/3	0.40
Recurrences/metastases (yes/no) (9 cases missing)	28/8	5/1	0.12	9/3	9/2	15/4	0.92	25/8	8/1	0.39
Site of metastases (lung/bone/others)	18/13/21	3/2/4	0.97	3/4/5	6/4/7	12/7/13	0.92	18/12/18	3/3/7	0.52
Status (NED-DOC/AWD-DOD) (2 cases missing)	14/28	1/6	0.41	7/6	2/11	6/17	0.08	12/27	2/8	0.50

*MMRp* mismatch repair proficient, *MMR* mismatch repair deficient, *wt* wild type, *M* male, *F* female, *NED* no evidence of disease, *DOC* died of other causes, *AWD* alive with disease, *DOD* died of disease

**Supplementary Table** 1 Genes covered by the Oncomine Comprehensive Assay v3 (Thermo Fisher Scientific, Waltham, MA, USA) panel

**Supplementary Table** 2 List and characteristics of the different bioinformatic tools used for variant classification

**Supplementary Table** 3 Molecular data obtained in NGS analysis using OCAv3 panel

**Supplementary Fig.** 1 Co-mutated genes in *RAS*-mutated and *TP53*-mutated cases belong to alternative molecular pathways. In light orange co-mutations exclusive in *RAS-*mutated cases, in light blue co-mutations exclusive in *TP53-*mutated cases, whereas light pink corresponds to co-mutations occurring in both molecular subgroups

## Discussion

Poorly differentiated thyroid carcinomas, classified as high-grade, follicular cell–derived, non-anaplastic thyroid carcinomas according to strict WHO criteria, were molecularly characterized with a specific focus on identifying alterations that may serve as potential therapeutic targets.

Part of the study was designed to assess the presence and prevalence of alterations in the MMR system. Data on MMR alterations in thyroid carcinomas are relatively scarce. In a study on 241 thyroid carcinomas with different histologies, 7.5% of cases showed loss of MMR proteins, including two cases of PDTC (with a prevalence of MMR deficiency in 4.7% of PDTC in the Authors’ series) [19]. Interestingly, the presence of MMR-deficiency or germline mutations in MMR genes in thyroid cancer have been significantly correlated with the occurrence of double primary cancers [20, 21]. In our series, 11.9% of cases presented a MMR-deficient immunophenotype, thus showing a prevalence higher than what expected in the overall thyroid cancer population. Moreover, other six cases have mutations in MMR genes, with an overall prevalence of 25.5% (13/51) of cases with an alteration affecting proteins and/or genes of the pathway. In terms of type of protein alterations, loss of MSH6 protein, alone or in combination with loss of MSH2, represented the most prevalent pattern, in line with the recent literature [19]. Microsatellite instability analysis using a panel clinically approved for colon and endometrial cancer, only, failed to detect profiles of instability in all protein-altered cases. This result strongly suggest that patterns of microsatellite instability are tumor-type specific and targeted panels based on real-time PCR developed for other cancer types may be not efficient to determine MMR defects in thyroid cancer [22]. As for the gene-to-protein correlation, 57.1% (4/7) of the cases with MMR deficiency at the protein level had mutations in MMR genes. Six other cases with MMR gene mutations had no altered protein profile, supporting that these mutations were either in heterozygosity or impaired protein functionality but not expression. Moreover, three cases with MMR-altered protein expression had no mutations in MMR genes. This observation supports the hypothesis that epigenetic regulation (i.e., promoter methylation) may be an alternative active mechanism of inactivation, as it has been described for *MLH1* in colorectal and endometrial cancer. However, this mechanism is not clearly described in the literature for *MSH6*, so far. Cases with MMR defects were not associated with any clinical or pathological feature.

DNA analysis through NGS testing using a wide targeted panel led the way in identifying three major molecular clusters: an *NRAS*-mutated cluster, a *TP53*-mutated cluster, and a non-*NRAS*/non-*TP53* cluster. *NRAS* mutations were mutually exclusive with *TP53* mutations. Key molecular features of the three clusters included the following:

- Non-*NRAS*/non-*TP53-*mutated cases were the most prevalent, with a mean number of mutations comparable with the *NRAS*-mutated group, and harboring *TERTp* mutations as the most frequent alteration (28%, 7/25);
- *NRAS*-mutated cancers had a low mean number of mutations, and the most frequent co-mutations occurred in *PIK3CA* and *TERTp* (23.1%, 3/13, each);
- *TP53*-mutated cancers had the highest mean number of mutations and were frequently co-mutated with *PTEN* (46.1%, 6/13) but lacked co-mutations in *TERTp*.

Interestingly, all but one immunohistochemically MMR-deficient tumors belonged to the *TP53*-mutated group. This result supports the hypothesis that defects in the MMR-system are more likely sustaining molecular mechanisms of progression, rather than representing early driver alterations [23]. Interestingly, a hypermutated phenotype is a recurrent phenomenon in anaplastic thyroid carcinoma [24], and it has been associated with the presence of MMR defects [25]. Starting from the observations that, in our series, MMR defects were more prevalent in the *TP53*-mutated group and that this molecular group also showed the highest rate of mutations, it is reasonable to hypothesize that this subgroup bears, as compared to the other two subgroups, a molecular profile closer to the one of anaplastic carcinoma.

This overall scenario is in line with some previous literature data. In particular, our data strongly support that the PDTC subtype—as defined by Turin consensus criteria—is separate even molecularly from the other high-grade differentiated thyroid cancers, mainly because of the high prevalence of *NRAS* mutations and the extremely low prevalence of *BRAF* mutations [10, 26, 27]. Interestingly, the three *BRAF* mutations detected in our series were not of the *BRAF*-p.V600E type, thus further supporting a dominant *RAS*-like molecular profile of PDTC. Moreover, the mutually exclusive presence of *NRAS* and *TP53* mutations was already present in the recent study by Xu et al. [11], although with a different prevalence of mutations. However, *NRAS* and *TP53* mutations have been described to co-occur in PDTC in other studies [28] and *RAS*-like mutations (i.e., *BRAF* non-V600E mutations) were detected in *TP53*-mutated cases of our series. Therefore, the mutual exclusivity of *NRAS* and *TP53* observed in our study has not enough strength to support the existence of two distinct molecular subgroups, but has to be confirmed by future studies in independent case series.

Finally, we observed an overall prevalence of *TERTp* mutations (all validated by Sanger sequencing) lower than that of previous studies, and with a lower concurrence with *NRAS* mutations [11]. In this respect, a possible limitation of our study in detecting *TERTp* mutations is related to the low sensitivity of Sanger sequencing, as compared to highly sensitive techniques such as digital droplet PCR, that might have failed to detect cases, additional to those detected by means of NGS, harboring sub-clonal variants.

The three PDTC molecular subgroups were not associated with peculiar clinical or pathological characteristics except for the presence of predominant oncocytic features, which was more prevalent in the *TP53*-mutated group, as opposed to *NRAS*-mutated tumors. In terms of outcome and disease-free and disease-specific survivals, the three groups did not differ significantly. *TP53*-mutated and non-*NRAS*/non-*TP53-*mutated groups showed a higher proportion of cases with adverse outcome (alive with disease status or death because of cancer), but survival analyses failed to reach statistical significance. Therefore, we could not confirm the adverse impact on survival of *TP53* and *TERTp* mutations observed by Xu et al. [11]. However, this is most probably related to the fact that PDTC cases only, and no other high-grade differentiated carcinomas, were included in our study.

The lack of correlation between molecular groups and clinicopathological findings raises questions about the biological and clinical relevance of the molecular subgrouping and highlights a main limitation of this part of the study which is related to the relatively small sample size of each molecular group. Multiple comparison tests or more detailed survival analyses (i.e., multivariable tests) could not be performed due to the limited number of cases available. Moreover, the molecular characterization was limited to targeted sequencing of cancer-related genes and fusion analysis, without transcriptomic profiling. Therefore, integration of broader transcriptomic or epigenetic data would likely be necessary to support such subclassification with greater confidence.

In the detection of gene fusions by RNA-targeted sequencing, the prevalence of fusions previously reported in thyroid cancer was low (4.8%, 2/42) but consistent with previous data. Interestingly, the case harboring the *CCDC6::RET* fusion was also co-mutated in *NRAS*, thus raising the question whether this case would belong to a *RAS*-like or a *BRAF*-like genotype. However, it cannot be excluded that the coexistence of *NRAS* and *RET* alterations in this case is due to the presence of heterogeneous tumor populations derived from two distinct tumor foci colliding in the same lesion, a possibility well described in the literature [29]. More interestingly, two cases harbored the *TBL1XR1::PIK3CA* fusion, a molecular alteration never described in thyroid cancer, so far. *TBL1XR1* (Transducin beta-like 1X related protein 1, also known as TBLR1) encodes for a protein that acts as an integral subunit of the NCoR (nuclear receptor corepressor) and SMRT (silencing mediator of retinoic acid and thyroid hormone receptors) repressor complexes [30]. *TBL1XR1* mRNA is highly expressed in many human tissues, including thyroid, prostate, and breast tissues, and may function as an oncogene by activating many signal transduction pathways, such as Wnt-β-catenin, NF-κB, and Notch [31]. Rearrangement of *TBL1XR1* (3q26.32) have been identified in various cancers involving different genes, including *RARA* (17q21) [32], *HMGA1* (6p21) [33], *TP63* (3q28) [34], *RET* (10q11.2) [35], and *PIK3CA* (3q26.32) [36, 37]. In the case of *TBL1XR1::PIK3CA* fusion, the first exon of *TBL1XR1* is fused with the second exon of *PIK3CA* by inversion and leads to the complete transcription of the wild-type sequence of *PIK3CA* in the fusion transcript. *TBL1XR1* is thought to regulate the expression of nuclear hormone receptor co-repressor [38], and tissue types in which the *TBL1XR1::PIK3CA* fusions were found (invasive breast carcinoma and prostate cancer) are hormonally regulated [36, 39, 40]. Furthermore, *TBL1XR1::PIK3CA* fusions were detected in chordoma and pancreatic cancer [37, 41]. The recurrence of this alteration in our series supports the potential role of the *TBL1XR1::PIK3CA* fusion as a novel additional driver event in PDTC. The interest for this recurrent molecular event is also associated with its potential role as a druggable target for therapy, as suggested in other cancer models [37]. However, the oncogenic activity of *TBL1XR1::PIK3CA* fusion is not supported by in silico modeling or in vitro functional studies. Therefore, further experimental validation to determine its biological significance and potential therapeutic relevance is needed to characterize this alteration as a definitive driver event.

Apart from the impact of our results in the understanding of the pathogenesis of PDTC, the translational relevance of our data into the clinics is highlighted by two main aspects. The first is the high prevalence of MMR defects in PDTC that paves the way for clinical studies testing the potential benefit of immunotherapy specifically in these tumors, as recently suggested for anaplastic thyroid cancer [42]. Second, a relevant number of cases harbored mutations in potentially druggable genes, mainly coding for tyrosine kinases (i.e., *PDGFRA* and *PDGFRB*, *MET*, *EGFR*, *ERBB3*, *FGFR1*, and *FGFR2*) or involved in tyrosine kinases pathway regulation (i.e., *PIK3CA*). Although such mutations were individually rare (from 2 to 7% of cases), 31.4% (16/51) of patients had at least one of such targetable alterations, thus supporting a role of tyrosine kinase inhibitors in the future clinical scenario of PDTC patients, especially when poorly responsive or progressive along radio-iodine treatment. Preclinical data on the effective activation of tyrosine kinase pathways in thyroid cancer cells further support this hypothesis [43, 44].

In conclusion, PDTC in our series homogeneously classified by the Turin consensus were genomically clustered into *NRAS*-mutated tumors (with low mutational burden and co-mutations affecting genes involved in the same pathway), *TP53*-mutated cancers (with high mutational burden, absence of *TERTp* mutations, strong association with MMR defects, and predominant oncocytic features), and a third heterogeneous group of non-*NRAS*/non-*TP53* mutated cases. Overall, currently or potentially targetable gene fusions have a prevalence of 9.5% (4/42), including the *TBL1XR1::PIK3CA* fusion that has never been described in the thyroid, so far, thus increasing the number of driver alterations and possible therapeutic targets for this aggressive disease. Finally, 47% (24/51) of cases overall, in the group of cases with DNA data available, harbor mutations in genes involved in tyrosine kinases pathways potentially targetable and/or have defects in the MMR pathway that claim a high prevalence of cases candidates for target therapies including immunotherapy.

## Supplementary Information

Below is the link to the electronic supplementary material.

ESM 1 (24.5 MB)

ESM 2 (DOCX 15.2 KB)

ESM 3 (XLSX 16.4 KB)

ESM 4 (XLSX 29.9 KB)

## Data Availability

The datasets used and/or analyzed during the current study are available from the corresponding author on reasonable request.
