# Supplementary figures and images for: High Prevalence of Potential Molecular Therapeutic Targets in Poorly Differentiated Thyroid Carcinoma
Source: Endocr Pathol. 2025 Oct 22;36(1):38. doi: 10.1007/s12022-025-09883-y (PMC12546271; doi:10.1007/s12022-025-09883-y)

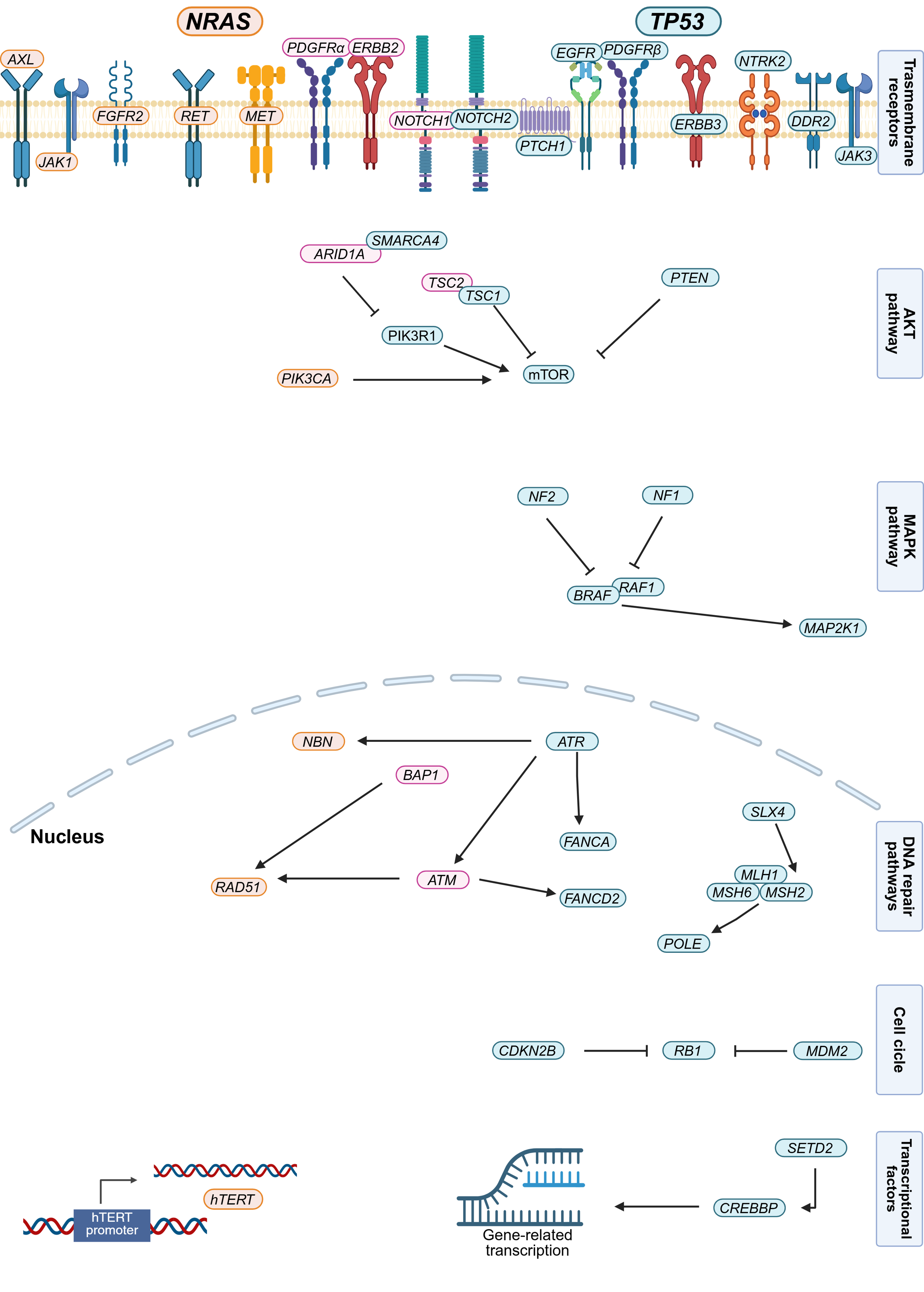

Supplement: Supplementary file 1 — (24.5 MB) [file 12022_2025_9883_MOESM1_ESM.tif]
