# Supplementary material for: High Prevalence of Potential Molecular Therapeutic Targets in Poorly Differentiated Thyroid Carcinoma
Source: Endocr Pathol. 2025 Oct 22;36(1):38. doi: 10.1007/s12022-025-09883-y (PMC12546271; doi:10.1007/s12022-025-09883-y)
Supplement: Supplementary file 2 — (DOCX 15.2 KB) [file 12022_2025_9883_MOESM2_ESM.docx]

**Supplementary Table 1**. Genes covered by the Oncomine™ Comprehensive Assay v3 (Thermo Fisher Scientific, Waltham, MA, USA) panel.

| Hotspot genes (87) | | | | Copy number variants (43) | | Fusion drivers (51) | | | Full exon coverage (48) | | |
| --- | --- | --- | --- | --- | --- | --- | --- | --- | --- | --- | --- |
| AKT1 | ESR1 | KIT | PDGFRB | AKT1 | FGFR4 | AKT2 | KRAS | RB1 | ARID1A | NF1 | STK11 |
| AKT2 | EZH2 | KNSTRN | PIK3CA | AKT2 | FLT3 | ALK | MDM4 | RELA | ATM | NF2 | TP53 |
| AKT3 | FGFR1 | KRAS | PIK3CB | AKT3 | IGF1R | AR | MET | RET | ATR | NOTCH1 | TSC1 |
| ALK | FGFR2 | MAGOH | PPP2R1A | ALK | KIT | AXL | MYB | ROS1 | ATRX | NOTCH2 | TSC2 |
| AR | FGFR3 | MAP2K1 | PTPN11 | AR | KRAS | BRAF | MYBL1 | RSPO2 | BAP1 | NOTCH3 |  |
| ARAF | FGFR4 | MAP2K2 | RAC1 | AXL | MDM2 | BRCA1 | NF1 | RSPO3 | BRCA1 | PALB2 |  |
| AXL | FLT3 | MAP2K4 | RAF1 | BRAF | MDM4 | BRCA2 | NOTCH1 | TERT | BRCA2 | PIK3R1 |  |
| BRAF | FOXL2 | MAPK1 | RET | CCND1 | MET | CDKN2A | NOTCH4 |  | CDK12 | PMS2 |  |
| BTK | GATA2 | MAX | RHEB | CCND2 | MYC | EGFR | NRG1 |  | CDKN1B | POLE |  |
| CBL | GNA11 | MDM4 | RHOA | CCND3 | MYCL | ERBB2 | NTRK1 |  | CDKN2A | PTCH1 |  |
| CCND1 | GNAQ | MED12 | ROS1 | CCNE1 | MYCN | ERBB4 | NTRK2 |  | CDKN2B | PTEN |  |
| CDK4 | GNAS | MET | SF3B1 | CDK2 | NTRK1 | ERG | NTRK3 |  | CHEK1 | RAD50 |  |
| CDK6 | H3F3A | MTOR | SMAD4 | CDK4 | NTRK2 | ESR1 | NUTM1 |  | CREBBP | RAD51 |  |
| CHEK2 | HIST1H3B | MYC | SMO | CDK6 | NTRK3 | ETV1 | PDGFRA |  | FANCA | RAD51C |  |
| CSF1R | HNF1A | MYCN | SPOP | EGFR | PDGFRA | ETV4 | PDGFRB |  | FANCD2 | RAD51D |  |
| CTNNB1 | HRAS | MYD88 | SRC | ERBB2 | PDGFRB | ETV5 | PIK3CA |  | FANCI | RAD51B |  |
| DDR2 | IDH1 | NFE2L2 | STAT3 | ESR1 | PIK3CA | FGFR1 | PPARG |  | FBXW7 | RB1 |  |
| EGFR | IDH2 | NRAS | TERT | FGF19 | PIK3CB | FGFR2 | PRKACA |  | MLH1 | RNF43 |  |
| ERBB2 | JAK1 | NTRK1 | TOP1 | FGF3 | PPARG | FGFR3 | PRKACB |  | MRE11 | SETD2 |  |
| ERBB3 | JAK2 | NTRK2 | U2AF1 | FGFR1 | RICTOR | FGR | PTEN |  | MSH2 | SLX4 |  |
| ERBB4 | JAK3 | NTRK3 | XPO1 | FGFR2 | TERT | FLT3 | RAD51B |  | MSH6 | SMARCA4 |  |
| ERCC2 | KDR | PDGFRA |  | FGFR3 |  | JAK2 | RAF1 |  | NBN | SMARCB1 |  |
